# ATPP: A Pipeline for Automatic Tractography-Based Brain Parcellation

**DOI:** 10.3389/fninf.2017.00035

**Published:** 2017-05-29

**Authors:** Hai Li, Lingzhong Fan, Junjie Zhuo, Jiaojian Wang, Yu Zhang, Zhengyi Yang, Tianzi Jiang

**Affiliations:** ^1^Brainnetome Center, Institute of Automation, Chinese Academy of SciencesBeijing, China; ^2^National Laboratory of Pattern Recognition, Institute of Automation, Chinese Academy of SciencesBeijing, China; ^3^University of Chinese Academy of SciencesBeijing, China; ^4^Key Laboratory for NeuroInformation of the Ministry of Education, School of Life Science and Technology, University of Electronic Science and Technology of ChinaChengdu, China; ^5^CAS Center for Excellence in Brain Science and Intelligence Technology, Institute of Automation, Chinese Academy of SciencesBeijing, China; ^6^Queensland Brain Institute, The University of QueenslandBrisbane, QLD, Australia

**Keywords:** parcellation, brain atlas, neuroimaging pipeline, diffusion tractography, parallel computing

## Abstract

There is a longstanding effort to parcellate brain into areas based on micro-structural, macro-structural, or connectional features, forming various brain atlases. Among them, connectivity-based parcellation gains much emphasis, especially with the considerable progress of multimodal magnetic resonance imaging in the past two decades. The Brainnetome Atlas published recently is such an atlas that follows the framework of connectivity-based parcellation. However, in the construction of the atlas, the deluge of high resolution multimodal MRI data and time-consuming computation poses challenges and there is still short of publically available tools dedicated to parcellation. In this paper, we present an integrated open source pipeline (https://www.nitrc.org/projects/atpp), named Automatic Tractography-based Parcellation Pipeline (ATPP) to realize the framework of parcellation with automatic processing and massive parallel computing. ATPP is developed to have a powerful and flexible command line version, taking multiple regions of interest as input, as well as a user-friendly graphical user interface version for parcellating single region of interest. We demonstrate the two versions by parcellating two brain regions, left precentral gyrus and middle frontal gyrus, on two independent datasets. In addition, ATPP has been successfully utilized and fully validated in a variety of brain regions and the human Brainnetome Atlas, showing the capacity to greatly facilitate brain parcellation.

## Introduction

From the well-known Brodmann atlas (Brodmann, 1909), which was released over 100 years ago, to the recently published Brainnetome Atlas (Fan et al., 2016) and HCP parcellation (Glasser et al., 2016), brain parcellations or atlases are in transition from purely *ex vivo* histology-based printed atlases to powerful neuroimaging-based digital brain maps with multimodal *in vivo* information. Massive and continuous efforts to parcellate the brain into areas have been made based on micro-structural, macro-structural or connectional features (Toga et al., 2006; Amunts and Zilles, 2015). Early parcellation efforts aimed at defining regional boundaries relied on post-mortem macro- or micro-architecture using limited number of samples. In the past two decades, information extracted from advanced brain mapping technologies, in particular multimodal magnetic resonance imaging (MRI), including structural, functional, and diffusion-weighted MRI, has offered alternative ways to tackle the challenge of cortical cartography (Fan et al., 2016).

Among them, connectivity-based parcellation has gained more and more weights in the community. A considerable number of studies have already used connectivity-based parcellation to form cartographic maps of specific regions of the brain or the entire cortex (Behrens et al., 2003; Johansen-Berg et al., 2004; Cohen A. L. et al., 2008; Cohen M. X. et al., 2008; Kim et al., 2010; Eickhoff et al., 2011; Chen et al., 2012; Craddock et al., 2012; Moreno-Dominguez et al., 2014; Fan et al., 2016; Glasser et al., 2016). It is a well-accepted concept that each cortical area having a unique pattern of inputs and outputs (“connectional fingerprint”), together with the local infrastructure characterized by micro-structural properties, represents the major determinant of the function of that area (Passingham et al., 2002). Connectivity-based parcellation is based on the assumption that those voxels/vertices belonging to a given brain area share similar connectivity profiles, characterized by structural (Behrens et al., 2003; Cohen M. X. et al., 2008; Moreno-Dominguez et al., 2014), functional (Cohen M. X. et al., 2008; Kim et al., 2010; Craddock et al., 2012), or meta-analytic connectivity (Eickhoff et al., 2011; Yang et al., 2016), as well as genetic correlation (Chen et al., 2012; Cui et al., 2016). In turn, brain areas should thus be definable by aggregating voxels/vertices showing similar connectivity patterns into larger clusters.

The Brainnetome project was launched to investigate the hierarchy in the human brain from genetics to neuronal circuits to behaviors (Jiang, 2013), conceptualizing the two components (nodes and connections) forming networks as the basic research unit. One of the main goals of the Brainnetome is to set up and optimize the framework for connectivity-based brain parcellation, and to produce a new human brain atlas. The resulting human Brainnetome Atlas (Fan et al., 2016), delineating 210 cortical and 36 subcortical subregions based on structural connectional architecture, is an *in vivo* atlas with not only more fine-grained functional subregions than traditional atlases but also connectional patterns of each area. The enriched region-specific information could help researchers to describe the locations of the activation or connectivity in the brain at much higher accuracy.

Structural connectivity-based parcellation for a specific brain region or the entire cortex, such as in the human Brainnetome Atlas, requires processing substantial amount of data, including high resolution multimodal MRI raw data and intermediate results. For instance, the volume of unprocessed raw data from recently released Human Connectome Project (Van Essen et al., 2012) S900 is nearly 12 TB. Besides, the computation consisting of multiple steps is time-consuming and error-prone, calling for efficient software engineering framework and automated algorithms. Both the data and the computational load pose challenges to researchers in the field. However, there is still short of available tools dedicated to parcellation in the community. In the course of building the human Brainnetome Atlas, we developed an integrated pipeline, named Automatic Tractography-based Parcellation Pipeline (ATPP), as an implementation of the framework of connectivity-based parcellation. ATPP features highly automated processing and massive parallel computing. It is conveniently scalable to run on desktop computers and high performance computing clusters, which is suitable for parcellating a specific region of interest (ROI) once or multiple ROIs simultaneously, respectively. ATPP has been successfully utilized and fully validated in parcellating a variety of brain regions (Xu et al., 2015; Genon et al., 2016; Zhang et al., 2016; Zhuo et al., 2016) and the human Brainnetome Atlas (Fan et al., 2016).

## Framework of ATPP

### Overview

**Figure 1:**
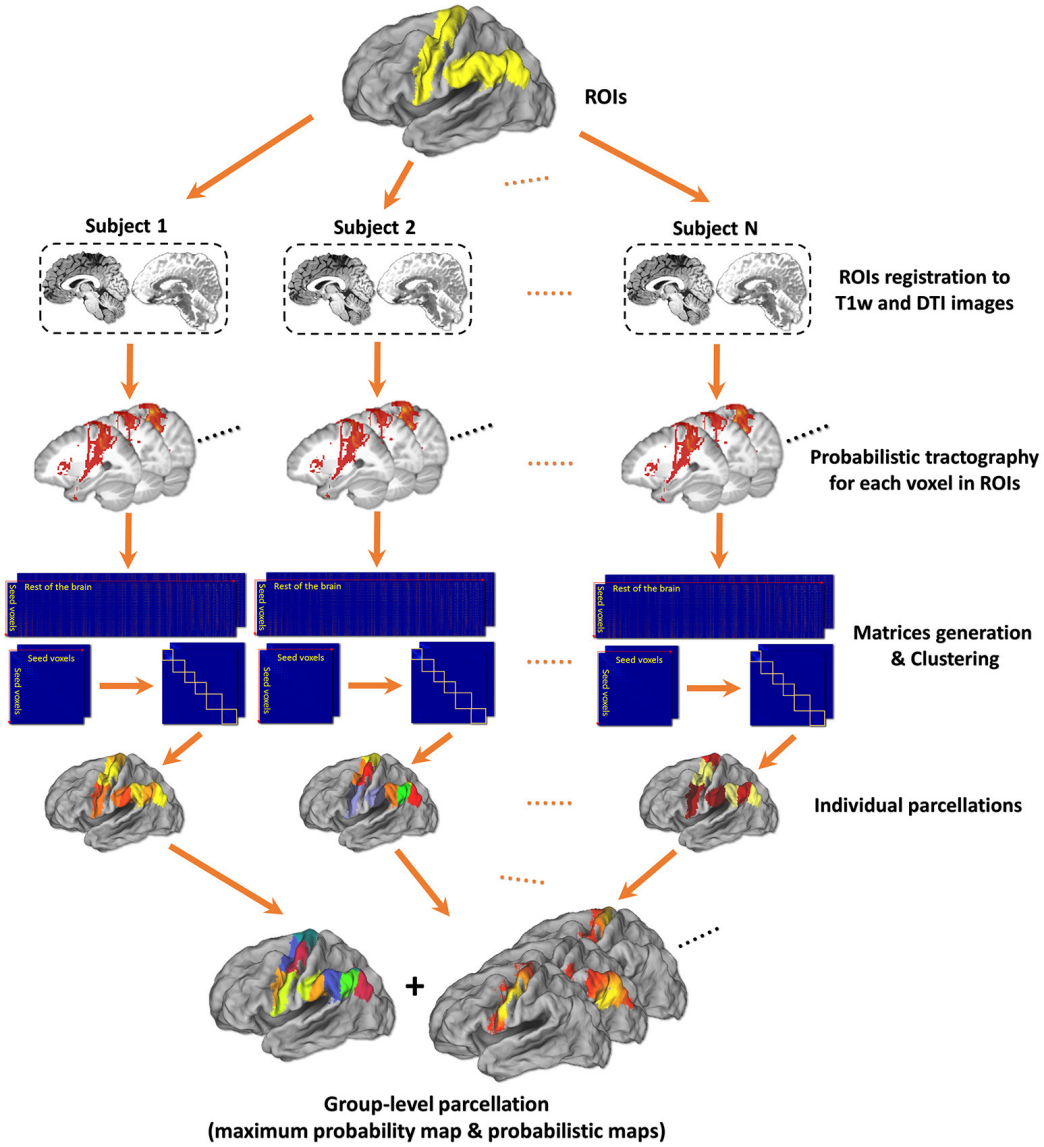
Framework of tractography-based brain parcellation. Based on T1w and DTI images of the same subjects, two given ROIs, left Precentral Gyrus (PrG) and left Inferior Parietal Lobule (IPL), are parcellated simultaneously. After a series of processing steps, mainly including registration, probabilistic tractography, matrix generation, and clustering, both the individual parcellations and the group-level parcellations of with a maximum probabilistic map and probabilistic maps of each subregion of left PrG and left IPL are produced.

The framework of tractography-based brain parcellation (Figure 1) accepts the defined ROI(s) and some parameters configured by users and automatically produce the final parcellation results with log information after a series of connected processing steps. Key steps are described in detail below.

### Registration

For each subject in a cohort, the skull-stripped T1-weighted image is co-registered to the corresponding non-diffusion-weighted images (*b* = 0 s/mm^2^, b0 images) using spatial parametric mapping (SPM8^1^), resulting in a co-registered T1 (rT1) images in the space of diffusion-weighted images. Then the rT1 images of the cohort are transformed to a standard template (e.g., MNI 152 structure template) using two-step spatial normalization, i.e., linear affine registration (Ashburner et al., 1997) and non-linear deformations (Ashburner and Friston, 1999), in SPM8. Finally, forward and inverse transformations between the individual diffusion space and the standard space are derived. Given the predefined ROI in a standard template, which is either extracted from a known atlas or drawn manually, an inverse transformation is performed to transform the ROI into a seed mask in the diffusion space for each subject. In addition, the forward transformation is used again in the subsequent step where the parcellated clusters of a seed mask in the diffusion space are transformed into the standard space.

### Probabilistic tractography

For each voxel in a seed mask, the probability distributions are estimated for multiple fiber directions (Behrens et al., 2007) using *bedpostx* tool. Probabilistic tractography is then applied by sampling many (e.g., 5,000, default value in *probtrackx*) streamlines to estimate the connectivity probability, resulting in an image file that represents each voxel's connectivity profile at whole-brain level. In such an image, the connectivity probability from the seed voxel *i* to another voxel *j* is defined by the number of streamlines passing through voxel *j* divided by the total number of streamlines sampled from voxel *i*. To compensate for the distance-dependent bias, probability counts are corrected by the length of the pathway (Tomassini et al., 2007). A small threshold value is used to threshold the path distribution estimates (e.g., connectivity probability value *p* > 0.04%, i.e., 2 out of 5,000 samples) (Makuuchi et al., 2009). By using this fixed threshold, the images not only have fewer false-positive connections (random noise), but also retain enough sensitivity to not miss true connections (Heiervang et al., 2006; Johansen-Berg et al., 2007).

### Individual parcellation

To facilitate data storage and analysis, the whole-brain connectivity profile at each voxel in a seed ROI is down-sampled (e.g., 5 mm isotropic voxels) (Johansen-Berg et al., 2004) and formed into a native connectivity matrix. Based on this matrix, a cross-correlation matrix between the connectivity profiles of all voxels in the seed mask is calculated and used for automatic parcellation. The *(i,j)th* element of the cross-correlation matrix is the correlation between the connectivity profile of seed *i* and the connectivity profile of seed *j* (Johansen-Berg et al., 2004). To define distinct clusters, the cross-correlation matrix is then processed using normalized-cut spectral clustering (Ng et al., 2001) without spatial constraint (Fan et al., 2014) to group voxels with similar connectivity profiles together. It should be noted that the number of clusters *k* must be determined by the experimenter when using this method. To facilitate making such decisions, *k* can be set as a range (e.g., from 2 to 12) in ATPP to generate multiple solutions in one go.

### Consistent relabeling

For each solution with the same *k* from different subjects, corresponding clusters are all warped into the standard template by the forward transformation produced previously. To resolve the cluster label mismatch issue caused by the random labeling of clustering algorithms across subjects, we try to find the most consistent labeling scheme across subjects by the following steps. First, the labeling schemes of all subject's clusters are pooled into a thresholded group-level cross-correlation matrix where each entry represents the connectional similarity of any two voxels in ROI. Then, the spectral clustering algorithm is applied again on this similarity matrix and a group-level labeling scheme is, thus, yielded. Last, the labeling scheme is propagated back to each subject's clusters by maximization of spatial overlap using an assignment algorithm (Munkres, 1957). In addition, Due to convergent evidences from different studies (Brodmann, 1909; Petrides and Pandya, 2002; Chen et al., 2012; Bludau et al., 2014; Cui et al., 2016) that support the topological homology across the hemispheres, if two ROIs representing the corresponding regions across hemispheres are given, the label consistency across hemispheres is ensured before propagation of the labeling scheme.

### Probabilistic maps and maximum probability maps

For each solution, the voxelwise probabilistic map of each cluster, i.e., the subregion of the ROI under parcellation, in the standard space is calculated. At each voxel, such a map represents the relative number of subjects classifying the voxel into the given cluster. Therefore, it indicates the inter-individual variability of that subregion, specifically, higher value at that voxel indicates lower inter-individual variability for that subregion. Furthermore, the maximum probability map (MPM) is created for each solution across all the subjects. The MPM is calculated by assigning each voxel in the standard space to the subregion in which it is most likely to be located. If two or more subregions show the same probability at a particular voxel, this voxel is assigned to the area with the highest probabilities averaged over the 26 voxels directly adjacent (Eickhoff et al., 2005). As a post-processing step, noisy voxels whose labels are different from the majority label of the 6-connected neighbors in the clusters, especially around the boundaries, are corrected (Wang et al., 2012).

### Validity indices

To avoid arbitrary choice of the number of subregions, ATPP offers various validity indices for determining *k* of the optimal solution. These indices are generally grouped according to the following three criteria: (1) *consistency across parcellations criterion*: Cramer's V (Hoel et al., 1947), Dice coefficient (Dice, 1945), normalized mutual information (Witten and Frank, 2005), and variation of information (Meila, 2003); (2) *consistency within parcellation criterion*: averaged silhouette value (Rousseeuw, 1987) and continuity index; (3) *consistency of topology criterion*: hierarchical index (Kahnt et al., 2012), and topological distance index (Tungaraza et al., 2015).

#### Consistency across parcellations criterion

To highlight the reproducibility of parcellation, the solution that yields optimal consistency across subjects is assumed to contain the optimal number of clusters. The first three indices (Cramer's V, Dice coefficient, and normalized mutual information) as aforementioned reflect the degree of cluster overlap between two parcellations. The forth index, variation of information, measures the amount of information lost and gained in changing between two parcellations. These indices are calculated on the following datasets generated using three resampling techniques: (1) *split-half*, where subjects are equally divided into two random groups with many (e.g., 100) repetitions, in each repetition, the MPMs of the two groups are used for calculation; (2) *pairwise*, for each pair of subjects, their parcellations are directly used for calculation; (3) *leave-one-out*, the parcellation of one subject and the MPMs of the remaining subjects are used. The calculation and meaning of the four indices are described in detail below.

##### Cramer's V (CV)

CV measures the strength of association between two parcellations. Given the frequency table *T* in which entry *T*_*ij*_ (*i* = *1…m; j* = *1…n*) represents the degree of overlap between two clusters *A*_*i*_ and *B*_*j*_ located in parcellation *A* and *B*, respectively. Then, the Cramer's V is calculated as follows:

(1) $$\begin{array}{r} V=\sqrt{\frac{\chi^{2}}{N\cdot min\left(m-1,n-1\right)}} \end{array}$$

where *N* is grand total of the frequency table and χ^2^ is the chi-squared statistic:

(2) $$\begin{array}{r} \chi^{2}=\sum_{i,j}\frac{{\left(T_{ij}-\frac{T_{i.}T_{.j}}{N}\right)}^{2}}{\frac{T_{i.}T_{.j}}{N}} \end{array}$$

CV has values in the interval [0, 1], where high values indicate good consistency with a value of 1 indicating a perfect match.

##### Dice coefficient (Dice)

Given the parcellation *A* and *B* with *k* clusters, then Dice coefficient:

(3) $$\begin{array}{r} Dice=\frac{1}{K}\sum_{i}^{K}\frac{2\left(A_{i}\cap B_{i}\right)}{\left|A_{i}|+|B_{i}\right|} \end{array}$$

is calculated to measure the similarity of two parcellations and it ranges between 0 and 1, with 1 indicating the same parcellation.

##### Normalized mutual information (NMI)

From the information theoretical perspective, the similarity between the two parcellations could be measured by the mutual information. Specifically, the mutual information quantifies the “amount of information” obtained about one parcellation through the other parcellation.

(4) $$\begin{array}{r} NMI\left(A,B\right)=2\frac{I\left(A;B\right)}{H\left(A\right)+H\left(B\right)}=2\frac{\sum_{i,j}T_{ij}log\frac{T_{ij}}{T_{i.}T_{.j}}\;}{-\sum_{i}T_{i.}logT_{i.}-\sum_{j}T_{.j}logT_{.j}} \end{array}$$

where *I(A;B)* is the mutual information between parcellation *A* and *B*, and *H(A)* and *H(B)* are the entropies of parcellation *A* and *B*, respectively. Here, we use [*H*(*A*) + *H*(*B*)]/2 for normalization to get a tight upper bound on the mutual information. The value of NMI ranges from 0 to 1, and the more similar to each other, the higher value is obtained.

##### Variation of information (VI)

The VI measures the amount of information lost and gained in changing between two parcellations, thus, indicating the stability of parcellations. The calculation of VI is described as:

(5) $$\begin{array}{r} VI\left(A,B\right)=H\left(A\right)+H\left(B\right)-2I\left(A;B\right) \end{array}$$

From the definition of VI, we can conclude that low VI values indicate high stability between two parcellations, and *vice versa*. It is worth noting that the upper limit value of VI is not 1 but *H*(*A*) + *H*(*B*). Moreover, when comparing two solutions with different number of clusters, VI is an intrinsically convenient and efficient index to determine a stable number of clusters. Several empirical confirmation of the stable number were recently proposed (Kelly et al., 2010; Kahnt et al., 2012; Bzdok et al., 2015), similarly, here in ATPP, the *k* clusters solution is considered stable when there is a considerable increase from *k* to *k* + 1 solution and there is no significantly increase from *k* − 1 to *k* solution.

#### Consistency within parcellation criterion

Intuitively, for an optimal solution of clusters, the clusters themselves should be widely separated (*separation*) and the voxels of each cluster should be as close to each other as possible (*compactness*). Here, we adopt two simple indices to depict separation and compactness.

##### Averaged silhouette value

The silhouette value for each voxel is a measure of how similar that voxel is to voxels in its own cluster, when compared to voxels in other clusters. The silhouette value for the *i*th voxel is defined as:

(6) $$\begin{array}{r} S_{i}=\frac{b_{i}-a_{i}}{max\left(a_{i},\;b_{i}\right)} \end{array}$$

where *a*_*i*_ is the average distance from the *i*th voxel to the other voxels in the same cluster, and *b*_*i*_ is the minimum average distance from the *i*th voxel to voxels in a different cluster. Then an averaged silhouette value across all voxels is obtained for a solution. The distance metric used here is cosine distance derived from the native connectivity matrix. The value ranges from −1 to 1, and the *k* solution with higher value compared to *k* − 1 solution seems to be a good solution.

##### Continuity index

We propose a simple index to depict the extent of how voxels connect to each other, i.e., continuity, within a cluster. The continuity index is the averaged proportion of the maximum continuum with 6/18/26-connected neighbors in a cluster. The value ranges from 0 to 1, with 1 indicating a solution where clusters are compact without any discrete voxels.

#### Consistency of topology criterion

An optimal solution for parcellation is also assumed to contain inherent consistent topological structure, which reflects the brain organization. The following two indices depict the consistency of topology to some extent.

##### Hierarchical index (HI)

HI reflects the hierarchical structure of the different solutions by the average probability that a given cluster in *k* solution has only one “parent-cluster” in *k* − 1 solution (Kahnt et al., 2012). For the *k* solution, HI is computed according to:

(7) $$\begin{array}{r} HI_{k}=\frac{1}{k}\sum_{i=1}^{k}\frac{\max_{j}\left(x_{ij}\right)}{\bar{x_{i}}} \end{array}$$

where $\bar{x_{\text{i}}}=\overset{k-1}{\underset{j=1}{\sum }}x_{ij}$, and for each *k, x* is a matrix whose elements *x*_*ij*_ reflect the number of voxels in cluster *j*_*i* = 1…*k*_ stemming from cluster *j*_*j* = 1…*k*−1_ in *k* − 1 cluster solution. HI = 1 means a perfect hierarchical structure.

##### Topological distance (TpD)

TpD specifically measures the similarity of the topological arrangement of putative homologous brain areas between hemispheres and across subjects. For a paired solution, in the matrix of each hemisphere, the *(i,j)* entry of denotes the number of voxels from regions *i* that are spatially in contact (26-nearest neighbors) with voxels from region *j* and each row of the matrix is normalized (Tungaraza et al., 2015). The TpD between the left and right given region per hemisphere is defined as the cosine distance of the two normalized matrices after vectoring them. The TpD score ranges from 0 to 1. A score close to 0 suggests that two hemispheres have similar topology.

#### Determination of the optimal *K* solution

There remains a great challenge to determine the optimal solution for brain parcellation, since the underlying clustering is inherently an ill-posed problem where the goal is to partition the data into some unknown number of clusters based on intrinsic information alone (Jain, 2010). While there is no ground-truth parcellation of human brain, the practical “optimal” solution emerges depending on the different aims of investigations, i.e., cluster validity criteria. ATPP offers various validity indices both in the form of text and graph from the above three different perspectives. Users are recommended to carefully investigate the trends of those indices, especially the local extrema (peaks and valleys) where the good solution for each index putatively exist (Kelly et al., 2010; Bzdok et al., 2015). The comprehensive optimal *k* solution is indicated by majority vote of those good solutions (Bzdok et al., 2015). Furthermore, we can make a comprehensive decision by combining the results from the above data-driven approaches with the findings from other modalities including, but not limited to, cyto-/myelo-architectonics, functional MRI, cross-species evidence (Eickhoff et al., 2015).

## Implementation of ATPP

### Overview

We implemented the workflow of tractography-based brain parcellation based on a series of in house Linux shell scripts and MATLAB (version R2009a or above, the MathWorks Inc.) functions, combining FMRIB's Diffusion Toolbox^2^ (FDT) included in FSL 5.0 and SPM8, both of which are well-known and widely used in the neuroimaging community. Specifically, FDT is used for probabilistic tractography, SPM8 is applied for image registration, and the rest of the functions are mainly implemented by in house MATLAB functions. All of these functional modules are glued together by Linux shell scripts into a hierarchical platform, called ATPP. ATPP utilizes Grid Engine (previously known as Sun Grid Engine (SGE), later owned by Oracle and now by Univa Corporation) and MATLAB Parallel Computing Toolbox™ (PCT) for parallel computing across and within machines. Both command line (CLI) version and graphical user interface (GUI) version are available. The CLI version is multi-ROI oriented and can be used to parcellate many brain regions simultaneously. While, the GUI version, designed by virtue of GTK-server^3^, is single-ROI oriented, and it is user-friendly to modify some parameters for parcellating a specific brain region.

**Figure 2:**
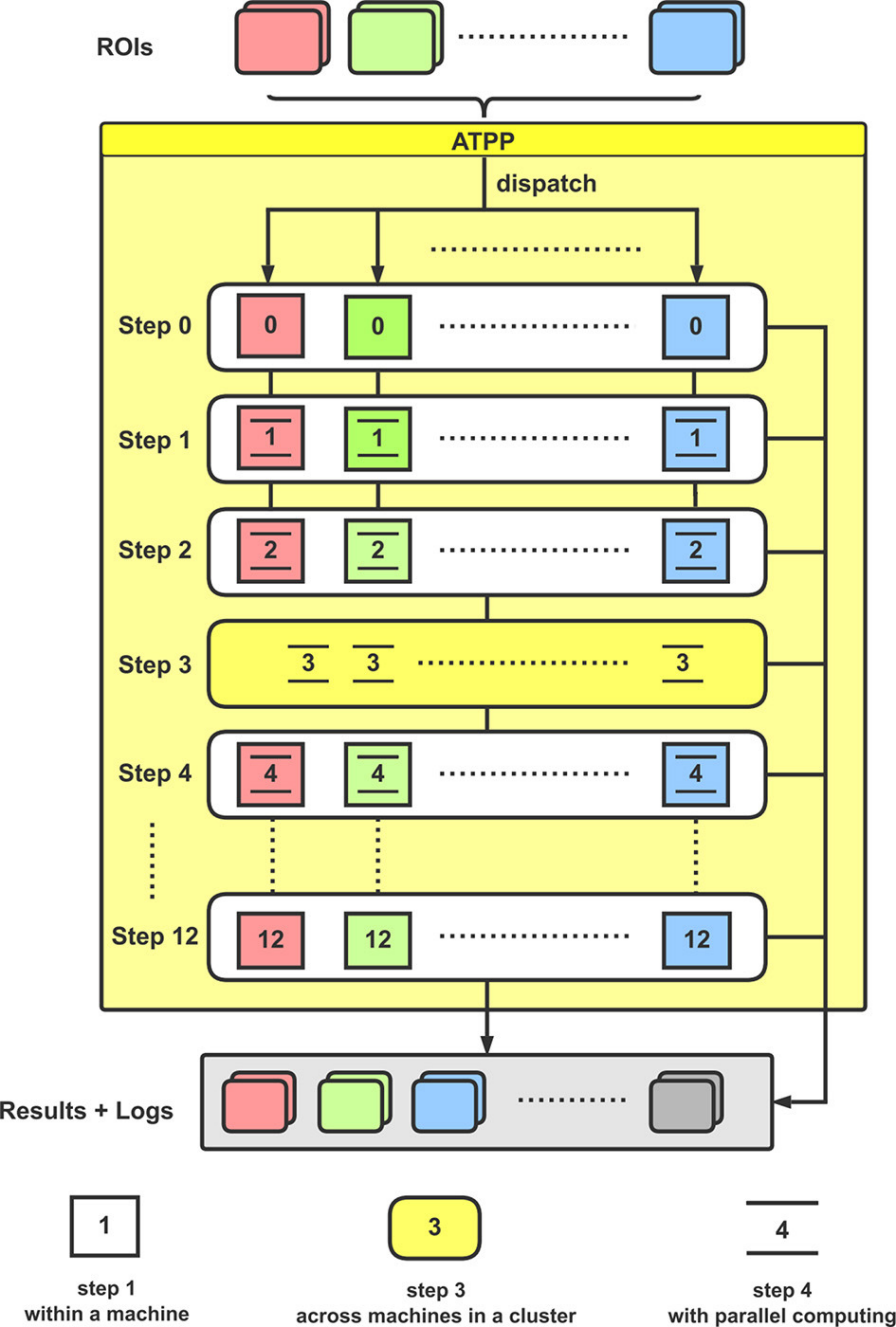
Flowchart for the implementation of ATPP. In a computer cluster, multiple given ROIs are distributed to different machines for executing a series of parcellation steps with paralleling computing within and across machines.

From the implementation point of view, the tractography-based brain parcellation pipeline is mainly split into the following steps (Figure 2):

0. The working directory and some essential files are generated.
1. ROI is registered from standard space to individual diffusion space.
2. For each registered ROI in diffusion space, a plain text which comprises the *xyz* coordinates of all non-zero voxels in the seed mask is generated.
3. Probabilistic tractography at each voxel in the registered ROIs is performed for each subject.
4. A cross-correlation matrix for each registered ROI is generated.
5. Clustering algorithm is applied in the cross-correlation matrix from the registered ROI.
6. The registered ROIs are inversely transformed from individual diffusion space to the standard space.
7. A consistent group-level labeling scheme is generated.
8. The labeling scheme is propagated back to individual parcellations for each subject.
9. Probabilistic maps for each subregion and the maximum probability map for each ROI across subjects are produced.
10. Some noise voxels of the MPM are removed.
11. Various validity indices are calculated.
12. The diagrams that depict the trends of various validity indices are produced.

With the given ROIs and configurations, ATPP can automatically process all the above 13 steps, which consist of registration, tractography, clustering, labeling, and validation, and accelerate the progress by massive parallel computing within and across machines. Eventually, the pipeline can not only generate parcellation results with different number of subregions and some validity indices, but also supply related processing logs for users to debug and examine the results.

### Prerequisites

Before running ATPP, users must check the following prerequisites. (1) *Input data*. ATPP requires skull-stripped T1-weighted (T1w) image and non-diffusion-weighted (b0) image as well as those images preprocessed by *bedpostx* (included in FSL) for each subject. (2) *Environment and tools*. Due to the programming language and dependencies of third-party programs, ATPP is designed to run on Linux operating system. There are several tools that are required to be installed in advance, such as FDT (included in FSL) and SPM8. In addition, for ATPP CLI version, SGE is required to be well configured. Other necessary tools are all included or integrated in the ATPP. In particular, the included GTK-server and related libraries need to be installed before running ATPP GUI version.

### Directory structure and file naming conventions

It is important for pipeline software to maintain simple, consistent, and scalable directory structure and file naming conventions. Without exception, ATPP has its own file and directory naming conventions. We commonly create an initial working directory for each ROI that contains: (1) a *ROI* subdirectory including the predefined ROIs, (2) a *log* subdirectory including the running logs, (3) *subject_id* subdirectories comprising T1w image and b0 image for each subject. There is an exemplar shell script in ATPP which is responsible for creating and organizing these working directories. A series of intermediate results and logs which have their specific and unified names will be generated during the running of pipeline.

### Hierarchical and modular structure of the implementation

The hierarchical structure of the implementation is initially inspired by the processing scripts of 1,000 Functional Connectome Project^4^. A top-level script (in CLI version) or callback functions (in GUI version), like the role of dispatchers, are responsible for reading configuration parameters and submitting jobs within or across machines. A second-level script, like the role of switchboard, is used to trigger a series of predefined steps and generate running logs. Third-level scripts are triggered to executing specific jobs either using in house MATLAB functions or third-party programs. The core algorithms implemented in each step are modular, thus can be easily and incrementally improved.

### Parallel computing

ATPP implements parallel computing across and within machines by means of SGE and MATLAB PCT, respectively. SGE is a job queuing system suitable for cluster computing or cloud computing that is in charge of scheduling, monitoring, and accounting jobs and load balancing. ATPP automatically distributes massive jobs via SGE to appropriate machines across the cluster. MATLAB PCT is toolbox that allows for executing code using multi-core processors with minimal modification to existing code. ATPP comprehensively utilizes PCT in the implementation code of each step to reduce the actual elapsed time.

### CLI implementation details

**Figure 3:**
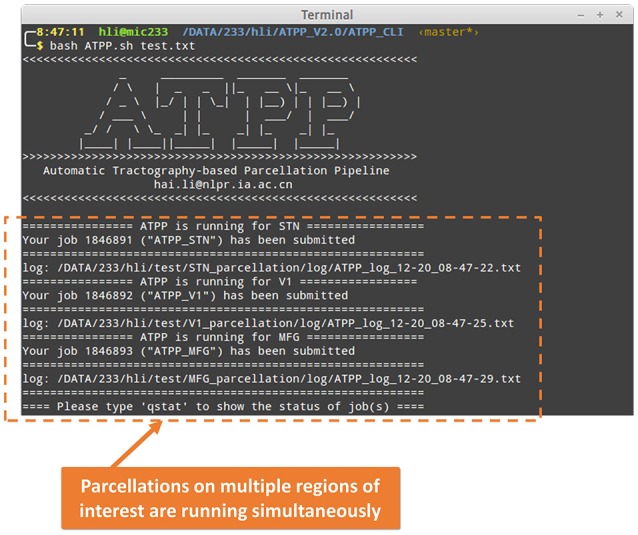
Command line (CLI) version of ATPP. CLI version is multi-ROI oriented, thus, users can parcellate multiple brain regions simultaneously. The figure shows a user *hli* submitted three concurrent tasks on parcellation of subthalamic nucleus (STN), primary visual cortex (V1), and middle frontal gyrus (MFG) at the same time.

ATPP CLI version (Figure 3) consists of a series of hierarchical bash shell scripts that glue in house MATLAB functions and/or third-party programs. Fed into a list file that defines the information (data directory, list of subjects, working directory, region name, and maximum number of subregions) of one region in each row, the top script, *ATPP.sh*, submits jobs that each contains a second-level script, *pipeline.sh*, and the information of one region as well as the configuration file, *config.sh*, to appropriate machines across the cluster. The second-level script triggers and logs a series of predefined third-level scripts, each representing a specific step, to execute specific tasks either using in house MATLAB functions or third-party programs according the configuration parameters. CLI version is multi-ROI oriented, thus is suitable for parcellating many regions simultaneously. It rests on a computing cluster, especially high performance computing cluster, and it is therefore efficient for the advanced users with projects that require processing multiple regions with massive computing.

### GUI implementation details

**Figure 4:**
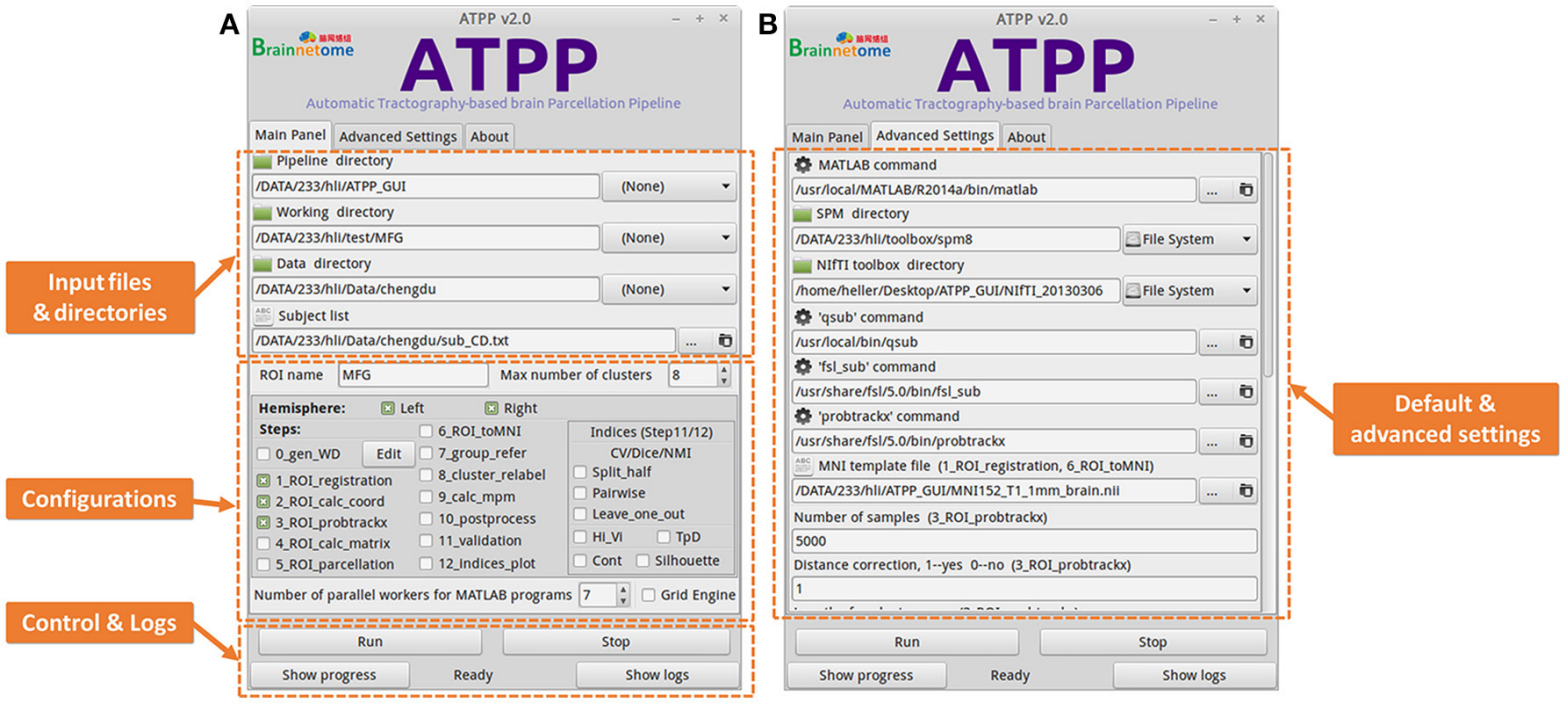
Graphical User Interface (GUI) version of ATPP. **(A)** The “Main Panel” tab includes indispensable and basic parameters including input files and directories as well as configuration parameters regarding to steps selection and parallel computing. **(B)** The “Advanced Settings” tab with advanced parameters including the paths of some commands and files as well as specific parameters in some steps. GUI version is single-ROI oriented, thus users can focus on a specific region and easily modify some parameters to test different processing conditions.

Some users with few programming skills prefer to a graphical panel that is easy-to-use and controls the whole running pipeline. ATPP GUI version (Figure 4) meets the demand. It is designed by virtue of GTK-server, an open source project that enables to access graphical user interfaces for shell scripts using GTK, to offer a user-friendly graphical panel. There are three tabs, the “Main Panel” tab with indispensable and basic parameters including input files and directories as well as configuration parameters regarding to steps selection and parallel computing, the “Advanced Settings” tab with advanced parameters including the paths of some commands and files as well as specific parameters in some steps, and the “About” tab with the information related to the developer and license, where users can input or modify various basic and advanced research-specific parameters. There is also a fixed area that contains buttons to allow users to control the startup and shutdown of jobs, triggering the status bar to circularly show “Ready,” “Running,” “Stop,” and “Done,” as well as examine the real-time running progress and detailed logs. Besides, ATPP GUI version offers parallel computing both within machine and across machines. Compared to CLI version, GUI version is single-ROI oriented, thus users can focus on a specific region and expediently modify some parameters to test different processing conditions.

## Results and discussion

In this study, we developed an integrated pipeline named ATPP realizing tractography-based brain parcellation with automatic processing and massive parallel computing. ATPP offers a powerful CLI version for parcellating multiple brain regions simultaneously and a user-friendly GUI version for parcellating a single brain region.

**Figure 5:**
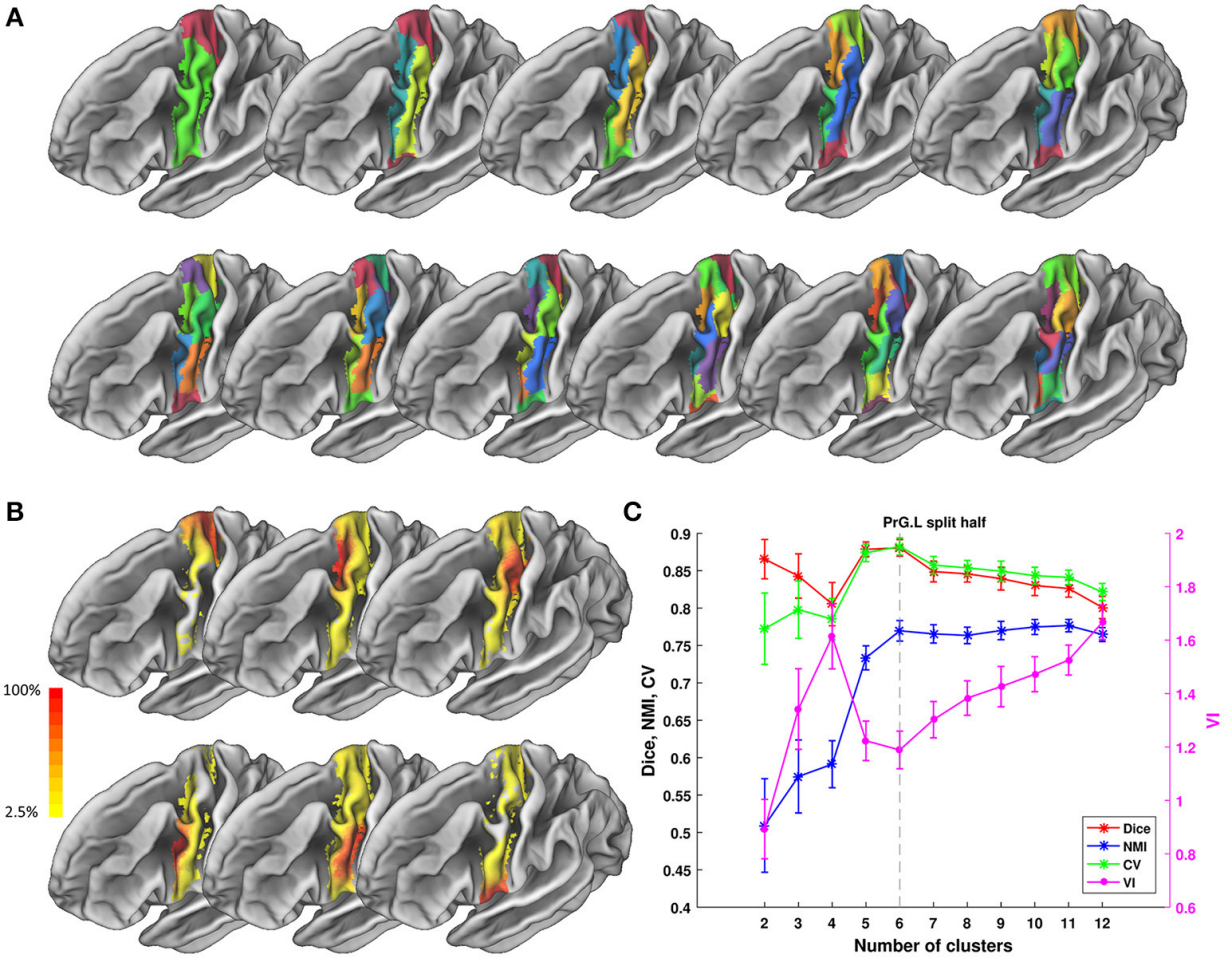
Parcellation results of left Precentral Gyrus (PrG.L) based on Chengdu data. **(A)** Maximum probability maps of PrG.L with 2-12 clusters solution. Note that there is no correspondence among subregions with the same color in different solution. **(B)** Probabilistic maps for each subregion in 6 clusters solution. The value of 1 indicates that the voxel belongs to the putative subregion across all subjects, i.e., there is low inter-subject variability at that voxel. Similarly, the lower values indicate higher inter-subject variability. **(C)** Validity indices of PrG.L in *split-half* resampling technique with 100 repetitions. The relative higher value of Dice, NMI, and CV and relative lower value of VI denote the more consistent parcellation across solutions. Error bars denote standard deviation. The optimal 6 clusters solution seems most reasonable according to those indices.

**Figure 6:**
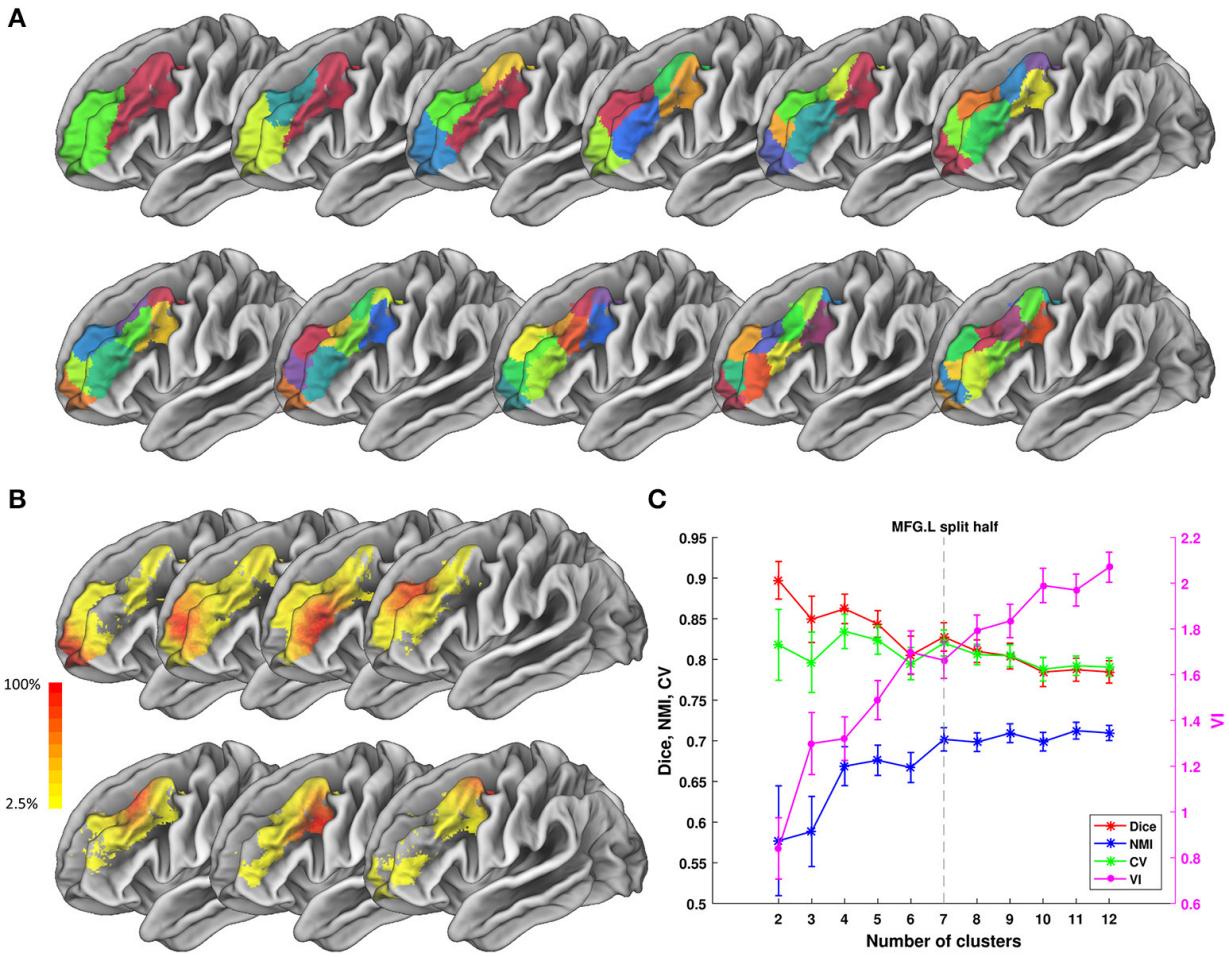
Parcellation results of left Middle Frontal Gyrus (MFG.L) based on HCP data. **(A)** Maximum probability maps of MFG.L with 2–12 clusters solution. Note that there is no correspondence among subregions with the same color in different solution. **(B)** Probabilistic maps for each subregion in 7 clusters solution. The value of 1 indicates that the voxel belongs to the putative subregion across all subjects, i.e., there is low inter-subject variability at that voxel. Similarly, the lower values indicate higher inter-subject variability. **(C)** Validity indices of MFG.L in *split-half* resampling technique with 100 repetitions. The relative higher value of Dice, NMI, and CV and relative lower value of VI denote the more consistent parcellation across solutions. Error bars denote standard deviation. The optimal 7 clusters solution shows most reasonable according to those indices.

We tested ATPP on two datasets in a local 10-node high performance computing cluster, where each node has 12 cores of Intel Xeon E5-2630@2.3 GHz and 128 GB memory. One dataset (Fan et al., 2016) has 40 normal participants (20 males; age range 17–20 years; diffusion MRI (dMRI) images with 2 mm isotropic voxels) recruited in Chengdu, China. The other dataset (Fan et al., 2016) has 40 normal subjects (18 males; age range 18–35 years; dMRI images with 1.25 mm isotropic voxels) selected from Human Connectome Project (HCP) Q1-Q3 data. The multimodal MRI data were preprocessed by the minimal preprocessing pipeline (Glasser et al., 2013). All subjects in Chengdu and HCP data provided written informed consent on forms approved by the Institutional Review Board of University of Electronic Science and Technology of China and Washington University in St. Louis, respectively. We used the GUI version of ATPP to parcellate left precentral gyrus (PrG) on Chengdu data and the CLI version of ATPP to parcellate left middle frontal gyrus (MFG) on HCP data. Figures 5, 6 shows the parcellation results of left PrG and left MFG, respectively, with optimal number of subregions and some stability indices. The time consumed of the entire pipeline was 30 h and nearly 114 h, respectively.

In general, from the perspective of implementation, there are two categories (Cui et al., 2015) of parallel workflow tools: (1) *flexible workflow tools* that allow users to customize automated workflows for any purpose, e.g., Laboratory of Neuro Imaging (LONI) Pipline (Rex et al., 2003), Java Image Science Toolkit (JIST) (Lucas et al., 2010), and Nipype (Gorgolewski et al., 2011); (2) *fixed workflow tools* that provide a completely established data processing workflow for a particular purpose, such as CIVET^5^, Configurable Pipeline for the Analysis of Connectomes^6^ (C-PAC), Pipeline for Analyzing braiN Diffusion imAges (PANDA) (Cui et al., 2013), Data Processing and Analysis for Brain Imaging (DPABI) (Yan et al., 2016). ATPP belongs to the second category. In some research fields, especially the rapidly developing connectivity-based parcellation, it is required that sufficient understanding on various concepts and algorithms, specific implementation details, and programming skills. A complete, ready-to-use, and optimized solution seems more suitable for interested users. Therefore, fixed workflow tools, like ATPP, exactly offer users dedicated and optimized solutions to focus on their research and offer developers more freedom to select and test appropriate components to some extent.

In recent years, a large number of studies related to connectivity-based parcellation were published, while there is still short of public parcellation tools in the community. pyClusterROI (Craddock et al., 2012) and SLIC (Wang and Wang, 2016) are tools dedicated to parcellating regions using resting-state functional MRI data, however, ATPP focuses on parcellation using diffusion MRI data with tractography. The constellation toolbox in BrainVISA^7^, which is not yet publicly released, is an implementation of groupwise parcellation using tractography on cortical surface (Lefranc et al., 2016), while ATPP is a publicly available implementation of volume-based parcellation at both individual-level and group-level. Until now, in contrast to the rich concepts and rapid progress of connectivity-based parcellation in these years, the number of available tools seems much fewer, partially because of a certain number of undocumented algorithms or inaccessible implementations. Due to an increasing number of neuroscientists, psychologists, or clinical investigators with few computational backgrounds devoting themselves to rapidly developing neuroimaging, publicly available and easy-to-use tools, e.g., parcellation workflow tools, deserve more attention in the community.

Compared to those existing parcellation tools, several advantages of ATPP arise. Above all, ATPP, to the best of our knowledge, is the first connectivity-based parcellation tool combined with massive parallel computing within and across machines, which has great advantages in the face of large volume of high resolution multimodal MRI raw and intermediate data and a large number of computing-intensive tasks. ATPP makes full use of available computing resources with whether pervasive multi-core desktop computers or multi-node high performance computing clusters which are increasingly popular in laboratories around the world. ATPP can greatly accelerate the reliable and reproducible research for users with more tests and validations due to the reduced computational time and effort. It has been extensively tested and greatly speeded up the construction of human Brainnetome Atlas.

Secondly, the modular structure of ATPP is easy to be modified and improved. In the current release, we realized the framework of tractography-based brain parcellation using selected modules, e.g., the registration accomplished by function modules in SPM8 and the clustering method realized by spectral clustering. With the rapid development of neuroimaging methods related to parcellation, these modules could be constantly upgraded or easily replaced by other implementations. In future versions, ATPP will add in modules with different implementations to provide more options for users, such as incorporating characteristics from other connectional modalities, implementing more clustering algorithms and validity indices. In addition, ATPP offers a user-friendly and continuously optimized GUI for users who prefer point-and-click interaction to command line operation.

Thirdly, plenty of intermediate results and abundant log information generated by ATPP play a critical role for users to control quality and increase reproducibility. Note that although ATPP fully automates all the processing steps, manual intervention, e.g., stopping and visually inspecting the intermediate results with unified and consistent names in some specific steps, is necessary for quality control to get correct or better results. For example, when after registration from one space to another space, it is strongly recommended that users carefully check the registered images and perform manual modification if necessary. In recent years, calls to improve the transparency and reproducibility of scientific research have risen in frequency and fervor (Nichols et al., 2016). During the running of ATPP, detailed logs including the executing hosts, the start and elapsed time, and abundant indication messages from core algorithms as well as the configuration files make users to easily reproduce findings with the same data processing and conveniently disseminate information.

Finally, ATPP completely follows the scientific cultural shift to open science, which aims at making scientific research including journal papers, lab notes, data, and, of course, workflow tools, accessible and transparent to all levels of society. ATPP is publicly accessible in Neuroimaging Informatics Tools and Resources Clearinghouse^8^ (NITRC) (https://www.nitrc.org/projects/atpp). Its source codes are hosted in GitHub^9^ (https://github.com/haililihai/ATPP_CLI; https://github.com/haililihai/ATPP_GUI), under the GNU generic purpose license version 3^10^ (GPLv3), and are welcome to download and fork. The Digital Object Identifiers (DOIs) providing a persistent way to make digital data easily and uniquely citable was created from Zenodo^11^ platform with those GitHub repositories (ATPP CLI v2.0.0, doi: https://doi.org/10.5281/zenodo.239702; ATPP GUI v2.0.0, doi: https://doi.org/10.5281/zenodo.239705). Besides, to promote Resource Identification Initiative (Bandrowski et al., 2016), which aims to promote research resource identification, discovery, and reuse, Research Resource Identifier (RRID) was curated (RRID:SCR_014815) by SciCrunch Resource Registry^12^ to avoid ambiguities on the tool name in addition to its version (Nichols et al., 2016).

The above features of ATPP make it a promising tool for brain parcellation. In addition to the application in some brain regions and the human Brainnetome Atlas, ATPP shows great capability to facilitate brain parcellation from various perspectives. For example, since the majority of already existed atlases were generated from an individual subject or a specific group of subjects, e.g., healthy adults in most cases, it is interesting to utilize ATPP to investigate the specific regions or atlases derived from those subjects with different age or suffering from a variety of psychological, neurodevelopmental, or neurodegenerative disorders. As another example, the slightly adapted version of ATPP with some modules replaced is also promising in the parcellation for non-human (e.g., primate) brain.

The current version of ATPP mainly focus on the implementation of structural connectivity-based parcellation for specific brain regions or the entire cortex. There are more connectional features, such as resting-state functional connectivity (Cohen A. L. et al., 2008; Kim et al., 2010), structural covariance (Cohen M. X. et al., 2008), meta-analysis-based functional co-activation (Eickhoff et al., 2011), and genetic correlation (Chen et al., 2012), in the framework of connectivity-based parcellation. Several studies indicate that resting-state connectivity (Honey et al., 2009; Van Den Heuvel et al., 2009) and meta-analytic co-activations (Eickhoff et al., 2010) reflect the underlying anatomical connectivity architecture of the human brain to some degree. Hence, in the future version of ATPP, whose modular structure make it easy to be modified and improved, it is an interesting and important direction to implement such multimodal connectivity-based parcellation. Moreover, these multimodal parcellations in turn contribute more information to the determination of optimal solution.

In summary, we developed an open source workflow tools named ATPP dedicated to tractography-based brain parcellation with automatic processing and massive parallel computing. Fully validated in the published parcellation of several brain regions, especially in the construction of the human Brainnetome Atlas, ATPP shows the capability to greatly facilitate brain parcellation.

## Author contributions

HL, LF, JZ, JW, YZ, ZY, and TJ were responsible for design and prototyping of the pipeline. HL, JW, and YZ were responsible for the implementation of the pipeline. HL did the test experiment of the pipeline. HL and ZY drafted this manuscript and all authors reviewed and approved the final version of the manuscript.

### Conflict of interest statement

The authors declare that the research was conducted in the absence of any commercial or financial relationships that could be construed as a potential conflict of interest. The handling Editor declared a shared affiliation, though no other collaboration, with one of the authors TJ and states that the process nevertheless met the standards of a fair and objective review.

## Footnotes

^1^http://www.fil.ion.ucl.ac.uk/spm/software/spm8

^2^http://fsl.fmrib.ox.ac.uk/fsl/fslwiki/FDT

^3^http://www.gtk-server.org/

^4^http://fcon_1000.projects.nitrc.org

^5^http://www.mcin-cnim.ca/neuroimagingtechnologies/civet/

^6^https://fcp-indi.github.io/

^7^http://www.brainvisa.info/web/index.html

^8^https://www.nitrc.org

^9^https://www.github.com

^10^https://www.gnu.org/licenses/gpl-3.0.html

^11^https://www.zenodo.org

^12^https://www.scicrunch.org/resources
